# 1-(4-Bromo­phen­yl)-3-(3,4-dimethyl­phen­yl)prop-2-en-1-one

**DOI:** 10.1107/S1600536810018106

**Published:** 2010-05-22

**Authors:** Yu-xia Zhou

**Affiliations:** a Shandong Vocational College of Science and Technology, Weifang 261061, People’s Republic of China

## Abstract

In the title chalcone derivative, C_17_H_15_BrO, the dihedral angle between the two benzene rings is 48.13 (4)°. In the crystal, a short Br⋯Br contact of 3.5052 (10) Å occurs.

## Related literature

For a related structure and background to chalcones, see: Fun *et al.* (2008 ▶).
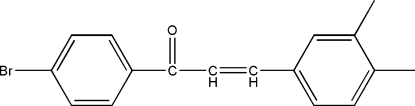


## Experimental

### 

#### Crystal data

C_17_H_15_BrO
*M* *_r_* = 315.20
Triclinic, 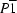

*a* = 5.9786 (14) Å
*b* = 7.8437 (19) Å
*c* = 15.744 (4) Å
α = 99.054 (4)°
β = 99.602 (4)°
γ = 95.659 (4)°
*V* = 713.0 (3) Å^3^
*Z* = 2
Mo *K*α radiation
μ = 2.87 mm^−1^
*T* = 298 K
0.25 × 0.22 × 0.20 mm

#### Data collection

Bruker SMART CCD diffractometer
3911 measured reflections
2620 independent reflections
2198 reflections with *I* > 2σ(*I*)
*R*_int_ = 0.019

#### Refinement

*R*[*F* ^2^ > 2σ(*F* ^2^)] = 0.033
*wR*(*F* ^2^) = 0.095
*S* = 1.09
2620 reflections
173 parameters
H-atom parameters constrained
Δρ_max_ = 0.45 e Å^−3^
Δρ_min_ = −0.60 e Å^−3^

### 

Data collection: *SMART* (Bruker, 1997 ▶); cell refinement: *SAINT* (Bruker, 1997 ▶); data reduction: *SAINT*; program(s) used to solve structure: *SHELXS97* (Sheldrick, 2008 ▶); program(s) used to refine structure: *SHELXL97* (Sheldrick, 2008 ▶); molecular graphics: *SHELXTL* (Sheldrick, 2008 ▶); software used to prepare material for publication: *SHELXTL*.

## Supplementary Material

Crystal structure: contains datablocks global, I. DOI: 10.1107/S1600536810018106/hb5445sup1.cif

Structure factors: contains datablocks I. DOI: 10.1107/S1600536810018106/hb5445Isup2.hkl

Additional supplementary materials:  crystallographic information; 3D view; checkCIF report

## Figures and Tables

**Fig. 1.**
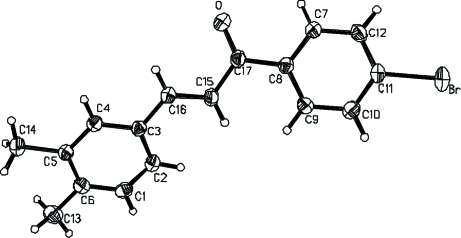
The molecular structure of (I) with displacement ellipsoids are drawn at the 30% probability level.

## supplementary crystallographic information

### Comment

As part of our search for new biologically active compounds we synthesized the title compound(I) and report its crystal structure herein.

In the crystal structure of compound(I)(fig.1),the dihedral angle between the two benzene rings(C1—C6) and (C7—C12) is 48.13 (4)°. All of the bond lengths and bond angles are in normal ranges and comparable to those in related structure (Fun *et al.*, 2008).

### Experimental

A mixture of 4-bromohypnone (0.02 mol) and 3,4-dimethylbenzaldehyde (0.02 mol) and 10%NaOH (5 ml) was stirred in ethanol (30 ml) for 1.5 h to afford the title compound (yield 73%).  Yellow blocks of (I) were obtailed by recrystallization from ethyl acetate at room temperature.

### Refinement

H atoms were positioned geometrically and allowed to ride on their parent atoms, with C—H distances of 0.93-0.96 Å, and with *U*_iso_(H) = 1.2*U*_eq_ of the parent atoms.

### Crystal data

**Table:**

C_17_H_15_BrO	*Z* = 2
*M**_r_* = 315.20	*F*(000) = 320
Triclinic, *P*1	*D*_x_ = 1.468 Mg m^−^^3^
Hall symbol: -P 1	Mo *K*α radiation, λ = 0.71073 Å
*a* = 5.9786 (14) Å	Cell parameters from 2198 reflections
*b* = 7.8437 (19) Å	θ = 1.3–25.5°
*c* = 15.744 (4) Å	µ = 2.87 mm^−^^1^
α = 99.054 (4)°	*T* = 298 K
β = 99.602 (4)°	Bar, yellow
γ = 95.659 (4)°	0.25 × 0.22 × 0.20 mm
*V* = 713.0 (3) Å^3^	

### Data collection

**Table:**

Bruker SMART CCD diffractometer	2198 reflections with *I* > 2σ(*I*)
Radiation source: fine-focus sealed tube	*R*_int_ = 0.019
graphite	θ_max_ = 25.5°, θ_min_ = 1.3°
phi and ω scans	*h* = −7→7
3911 measured reflections	*k* = −9→6
2620 independent reflections	*l* = −18→19

### Refinement

**Table:**

Refinement on *F*^2^	Secondary atom site location: difference Fourier map
Least-squares matrix: full	Hydrogen site location: inferred from neighbouring sites
*R*[*F*^2^ > 2σ(*F*^2^)] = 0.033	H-atom parameters constrained
*wR*(*F*^2^) = 0.095	*w* = 1/[σ^2^(*F*_o_^2^) + (0.048*P*)^2^ + 0.2458*P*] where *P* = (*F*_o_^2^ + 2*F*_c_^2^)/3
*S* = 1.09	(Δ/σ)_max_ < 0.001
2620 reflections	Δρ_max_ = 0.45 e Å^−^^3^
173 parameters	Δρ_min_ = −0.60 e Å^−^^3^
0 restraints	Extinction correction: *SHELXL97* (Sheldrick, 2008), Fc^*^=kFc[1+0.001xFc^2^λ^3^/sin(2θ)]^-1/4^
Primary atom site location: structure-invariant direct methods	Extinction coefficient: 0.048 (4)

### Special details

**Table:**

Geometry. All esds (except the esd in the dihedral angle between two l.s. planes) are estimated using the full covariance matrix. The cell esds are taken into account individually in the estimation of esds in distances, angles and torsion angles; correlations between esds in cell parameters are only used when they are defined by crystal symmetry. An approximate (isotropic) treatment of cell esds is used for estimating esds involving l.s. planes.
Refinement. Refinement of *F*^2^ against ALL reflections. The weighted *R*-factor wR and goodness of fit *S* are based on *F*^2^, conventional *R*-factors *R* are based on *F*, with *F* set to zero for negative *F*^2^. The threshold expression of *F*^2^ > σ(*F*^2^) is used only for calculating *R*-factors(gt) etc. and is not relevant to the choice of reflections for refinement. *R*-factors based on *F*^2^ are statistically about twice as large as those based on *F*, and *R*- factors based on ALL data will be even larger.

### Fractional atomic coordinates and isotropic or equivalent isotropic displacement parameters (Å^2^)

**Table:**

	*x*	*y*	*z*	*U*_iso_*/*U*_eq_	
Br	0.57731 (6)	0.43242 (4)	0.100602 (19)	0.06655 (18)	
C11	0.5449 (5)	0.3857 (3)	0.21288 (17)	0.0440 (6)	
O	0.2750 (3)	0.2761 (3)	0.48223 (13)	0.0628 (6)	
C10	0.7328 (5)	0.4220 (4)	0.27985 (18)	0.0482 (6)	
H10A	0.8724	0.4717	0.2706	0.058*	
C17	0.4645 (5)	0.2760 (4)	0.46327 (17)	0.0455 (6)	
C12	0.3349 (5)	0.3142 (4)	0.22550 (18)	0.0506 (7)	
H12A	0.2100	0.2898	0.1796	0.061*	
C9	0.7107 (4)	0.3834 (3)	0.36099 (17)	0.0448 (6)	
H9A	0.8374	0.4048	0.4061	0.054*	
C8	0.5010 (4)	0.3129 (3)	0.37593 (16)	0.0395 (5)	
C6	1.1464 (4)	0.0981 (3)	0.80706 (18)	0.0453 (6)	
C5	0.9589 (4)	0.1781 (4)	0.82839 (17)	0.0448 (6)	
C3	0.8289 (4)	0.1904 (3)	0.67426 (17)	0.0408 (6)	
C4	0.8024 (4)	0.2208 (3)	0.76150 (17)	0.0437 (6)	
H4A	0.6759	0.2714	0.7755	0.052*	
C16	0.6528 (5)	0.2356 (3)	0.60745 (17)	0.0437 (6)	
H16A	0.5194	0.2656	0.6256	0.052*	
C2	1.0206 (5)	0.1157 (3)	0.65442 (18)	0.0459 (6)	
H2A	1.0448	0.0967	0.5970	0.055*	
C7	0.3131 (5)	0.2796 (4)	0.30725 (17)	0.0464 (6)	
H7A	0.1717	0.2335	0.3167	0.056*	
C15	0.6631 (5)	0.2384 (4)	0.52402 (18)	0.0477 (6)	
H15A	0.7966	0.2164	0.5036	0.057*	
C1	1.1749 (5)	0.0697 (3)	0.72054 (19)	0.0474 (6)	
H1A	1.3008	0.0184	0.7064	0.057*	
C14	0.9249 (6)	0.2180 (5)	0.9221 (2)	0.0674 (9)	
H14A	0.7885	0.2726	0.9242	0.101*	
H14B	1.0541	0.2950	0.9567	0.101*	
H14C	0.9103	0.1119	0.9450	0.101*	
C13	1.3147 (6)	0.0413 (4)	0.8765 (2)	0.0635 (8)	
H13A	1.4312	−0.0106	0.8501	0.095*	
H13B	1.2362	−0.0421	0.9034	0.095*	
H13C	1.3838	0.1406	0.9201	0.095*	

### Atomic displacement parameters (Å^2^)

**Table:**

	*U*^11^	*U*^22^	*U*^33^	*U*^12^	*U*^13^	*U*^23^
Br	0.0949 (3)	0.0686 (3)	0.0421 (2)	0.01006 (17)	0.01910 (16)	0.02128 (15)
C11	0.0583 (16)	0.0408 (13)	0.0358 (13)	0.0098 (12)	0.0132 (12)	0.0091 (11)
O	0.0507 (12)	0.0955 (16)	0.0468 (11)	0.0114 (11)	0.0174 (9)	0.0161 (11)
C10	0.0477 (15)	0.0507 (15)	0.0470 (15)	0.0019 (12)	0.0133 (12)	0.0092 (12)
C17	0.0508 (16)	0.0485 (15)	0.0367 (13)	0.0058 (12)	0.0101 (11)	0.0043 (11)
C12	0.0514 (16)	0.0571 (17)	0.0394 (14)	0.0025 (13)	−0.0017 (12)	0.0104 (12)
C9	0.0420 (14)	0.0502 (15)	0.0387 (14)	0.0038 (11)	0.0027 (11)	0.0042 (11)
C8	0.0449 (14)	0.0387 (13)	0.0361 (13)	0.0085 (10)	0.0108 (10)	0.0052 (10)
C6	0.0446 (14)	0.0401 (13)	0.0501 (15)	0.0030 (11)	0.0045 (12)	0.0112 (12)
C5	0.0434 (14)	0.0507 (15)	0.0403 (14)	0.0011 (11)	0.0081 (11)	0.0106 (12)
C3	0.0434 (14)	0.0406 (13)	0.0395 (13)	0.0027 (10)	0.0096 (11)	0.0104 (11)
C4	0.0417 (14)	0.0500 (15)	0.0421 (14)	0.0088 (11)	0.0120 (11)	0.0098 (12)
C16	0.0459 (14)	0.0452 (14)	0.0413 (14)	0.0061 (11)	0.0113 (11)	0.0086 (11)
C2	0.0528 (15)	0.0460 (14)	0.0401 (14)	0.0052 (12)	0.0147 (12)	0.0052 (11)
C7	0.0423 (14)	0.0534 (16)	0.0428 (15)	0.0010 (12)	0.0067 (11)	0.0107 (12)
C15	0.0525 (16)	0.0562 (16)	0.0389 (14)	0.0138 (13)	0.0139 (12)	0.0120 (12)
C1	0.0432 (14)	0.0430 (14)	0.0580 (17)	0.0092 (11)	0.0149 (12)	0.0071 (12)
C14	0.0606 (19)	0.103 (3)	0.0422 (17)	0.0175 (17)	0.0120 (14)	0.0157 (17)
C13	0.0604 (19)	0.067 (2)	0.064 (2)	0.0181 (15)	0.0032 (15)	0.0181 (16)

### Geometric parameters (Å, °)

**Table:**

Br—C11	1.898 (3)	C3—C2	1.396 (4)
C11—C10	1.379 (4)	C3—C4	1.395 (4)
C11—C12	1.382 (4)	C3—C16	1.470 (4)
O—C17	1.219 (3)	C4—H4A	0.9300
C10—C9	1.382 (4)	C16—C15	1.329 (4)
C10—H10A	0.9300	C16—H16A	0.9300
C17—C15	1.480 (4)	C2—C1	1.389 (4)
C17—C8	1.495 (4)	C2—H2A	0.9300
C12—C7	1.381 (4)	C7—H7A	0.9300
C12—H12A	0.9300	C15—H15A	0.9300
C9—C8	1.391 (4)	C1—H1A	0.9300
C9—H9A	0.9300	C14—H14A	0.9600
C8—C7	1.396 (4)	C14—H14B	0.9600
C6—C1	1.387 (4)	C14—H14C	0.9600
C6—C5	1.400 (4)	C13—H13A	0.9600
C6—C13	1.510 (4)	C13—H13B	0.9600
C5—C4	1.393 (4)	C13—H13C	0.9600
C5—C14	1.512 (4)		
			
C10—C11—C12	121.5 (2)	C3—C4—H4A	118.9
C10—C11—Br	119.1 (2)	C15—C16—C3	127.2 (3)
C12—C11—Br	119.4 (2)	C15—C16—H16A	116.4
C11—C10—C9	119.1 (2)	C3—C16—H16A	116.4
C11—C10—H10A	120.5	C1—C2—C3	119.9 (2)
C9—C10—H10A	120.5	C1—C2—H2A	120.1
O—C17—C15	122.0 (3)	C3—C2—H2A	120.1
O—C17—C8	119.9 (2)	C12—C7—C8	120.7 (2)
C15—C17—C8	118.0 (2)	C12—C7—H7A	119.7
C11—C12—C7	119.1 (2)	C8—C7—H7A	119.7
C11—C12—H12A	120.5	C16—C15—C17	120.7 (3)
C7—C12—H12A	120.5	C16—C15—H15A	119.6
C10—C9—C8	120.8 (2)	C17—C15—H15A	119.6
C10—C9—H9A	119.6	C6—C1—C2	121.8 (2)
C8—C9—H9A	119.6	C6—C1—H1A	119.1
C9—C8—C7	118.9 (2)	C2—C1—H1A	119.1
C9—C8—C17	123.1 (2)	C5—C14—H14A	109.5
C7—C8—C17	117.9 (2)	C5—C14—H14B	109.5
C1—C6—C5	119.0 (2)	H14A—C14—H14B	109.5
C1—C6—C13	120.0 (3)	C5—C14—H14C	109.5
C5—C6—C13	121.0 (3)	H14A—C14—H14C	109.5
C4—C5—C6	118.9 (2)	H14B—C14—H14C	109.5
C4—C5—C14	120.0 (2)	C6—C13—H13A	109.5
C6—C5—C14	121.1 (3)	C6—C13—H13B	109.5
C2—C3—C4	118.2 (2)	H13A—C13—H13B	109.5
C2—C3—C16	123.1 (2)	C6—C13—H13C	109.5
C4—C3—C16	118.8 (2)	H13A—C13—H13C	109.5
C5—C4—C3	122.3 (2)	H13B—C13—H13C	109.5
C5—C4—H4A	118.9		
